# Test–retest reliability of four flatwater performance-related tests in canoe slalom athletes

**DOI:** 10.3389/fphys.2023.1277057

**Published:** 2023-11-01

**Authors:** Matej Vajda, Felix Krupa, Jan Busta, Jaylene Pratt

**Affiliations:** ^1^ Hamar Institute for Human Performance, Faculty of Physical Education and Sports, Comenius University, Bratislava, Bratislava, Slovakia; ^2^ Faculty of Physical Education and Sports, Comenius University, Bratislava, Slovakia; ^3^ Department of Swimming Sports, Faculty of Physical Education and Sport, Charles University, Prague, Prague, Czechia; ^4^ Auckland University of Technology, Auckland, Auckland, New Zealand

**Keywords:** canoeing, field-based testing, kayaking, performance-related fitness, white water

## Abstract

**Purpose:** This study aims to evaluate the test–retest reliability of four flatwater performance-related tests in canoe slalom athletes.

**Methods:** Twenty-two Slovak national team members of junior and U23 age group racing in a category K1 men (K1M), K1 women (K1W) or C1 men (C1M) volunteered to take part in this study. During both test and retest testing sessions athletes performed 4 flatwater tests: SPS—Sprints with a turn to the preferred side (2 × 15 m shuttle sprints), SNPS—Sprints with a turn to the non-preferred side (2 × 15 m shuttle sprints), SBS—Sprints with turns to both sides (2 × 15 m shuttle sprints) as well as 12 × 15 AOT—12 × 15 m all out shuttle test (12 × 15 m shuttle sprints). Each athlete completed two sessions separated by a minimum of days and a maximum of 5 days. Results: The results have shown the excellent test-retest reliability of all four flatwater tests (ICC—SPS: 0.98; SPNPS 0.97; SBS: 0.98 and 12 × 15 m AOT: 0.96). Additionally, results have shown SEM (SPS: 0.14; SPNPS 0.18; SBS: 0.13 and 12 × 15 m AOT: 1.05) and SWC (SPS: 0.21; SPNPS 0.26; SBS: 0.19 and 12 × 15 m AOT: 1.58). Conclusion: Based on our results we suggest that coaches use these valid and reliable tests to assess changes in their athletes’ performance-related physical fitness over time, to verify the effectiveness of training programs focused on improvement in specific physical fitness of athletes as well as to identify asymmetries between the preferred and non-preferred side in canoe slalom athletes.

## Introduction

Canoe slalom is one of the canoeing sports which is a regular part of the Olympic games since Barcelona 1992. The competition is organized on natural or artificial white-water courses specifically built for canoe slalom (ICF, 2022). Typical race duration varies in the range between 90 and 120 s and is dependent on the characteristics such as water level difficulties, the length of the course as well as the number of gates and their position (Nibali et al., 2011). Accordingly, to the rules of the International Canoe Federation (ICF) the course must consist of a minimum of 18 gates and a maximum of 25 gates, of which 6 or 8 must be upstream gates (at least 3 for each side). During the competition, the goal of the athletes is to navigate a boat through a combination of upstream and downstream gates (ICF, 2022). The competition includes four events for men´s kayak (K1M), men´s canoe (C1M), women´s kayak (K1W) and women´s canoe (C1W) and athletes are allowed to start in both kayak and canoe events (ICF, 2022).

There are various laboratory tests that were based on straightforward paddling in the pool or paddling on an ergometer and were proposed to assess the physiology of canoe slalom paddlers, such as aerobic power and/or anaerobic capacity. Messias et al. (2015), Ferrari et al. (2017), Busta et al. (2018), Bielik et al. (2019), Busta et al. (2019) These tests are regularly use to analyse physical fitness and physiological parameters of athletes but it is questionable if the tests which are based on straightforward paddling is optimal to analyses specific performance-related physical fitness in canoe slalom. As was previously mentioned by Zamparo et al. (2006) performance in canoe slalom is not only physiologically dependent but also highly technique dependent given that athletes must perform a series of turning maneuvers executed at high speed with changes in direction. These maneuvers are needed for the negotiation of upstream gates and the combination of downstream gates and are usually associated with a reduction in velocity or maintaining the position of a boat in the water (Macdermid et al., 2019). It should be noted that these turning maneuvers require higher force than straightforward paddling, and oxygen consumption is higher during flatwater paddling with turning maneuvers compared with straight-line paddling (Macdermid et al., 2019) Based on this information it is important to add turning maneuvers which led to specific physiological response to analyse performance-related physical fitness.

To date, only one test-retest study testing performance-related tests in canoe slalom was published by Baláš et al. (2020) who realized test-retest measurement of three flatwater tests of different distances (40, 80, and 200 m) with turning maneuvers. Authors found that these tests provide moderate to excellent reliability (ICC = 0.680–0.929) but these tests were performed without the gates and with pivoting turning maneuvers which not occurred on the competition runs. In practice, coaches mostly use simulated competitions that provide moderate reliability (Vieira et al., 2015) for on water testing. However, these simulated competitions are not under the standardized condition because courses and/or settings of the gates are changing over the season what does not allow to identify changes in performance-related physical fitness over time. Standardized flatwater tests for canoe slalom that include paddling and technical maneuvers are limited.

Thus, we decided to construct four flatwater tests which can be conducted in training practice, in a short space of time and can be completed by coaches without the need for highly sophisticated equipment. These tests are focused to establish performance-related physical fitness as well as asymmetry of the athletes.

This process was based on author’s experiences as a canoe slalom coach as well as on the discussion with other coaches and experts working in canoe slalom around the world. In the second step, we realized validation study (Vajda and Piatrikova, 2022) of constructed tests. The last step is to conduct reliability of these tests. Thus, the aims of the present study were therefore to explore and quantify the measurement reliability of the four flatwater tests.

## Methods

### Subjects

Twenty-two (n = 22) Slovak international-level canoe slalom athletes racing in a category K1 men (K1M), K1 women (K1W) or C1 men (C1M) volunteered to take part in this study (see Table 1).

**TABLE 1:** General characteristics of the athletes.

Values	K1M	K1W	C1M
	(n = 8)	(n = 7)	(n = 7)
age (y)	20.4 ± 2.3	19.6 ± 2.3	19.9 ± 2.1
heigh (cm)	178.8 ± 1.9	167.4 ± 7.6	181.3 ± 5.6
weight (kg)	72.4 ± 6.8	55.6 ± 5.1	73.3 ± 3.6
body muscle (kg)	38.0 ± 2.6	26.4 ± 2.8	38.2 ± 1.5
body fat (%)	8.5 ± 2.7	12.7 ± 1.3	7.9 ± 1.9
years of practice in canoe slalom	11.5 ± 2.1	10.3 ± 2.3	10.4 ± 2.2
number of training (sessions per week)	11.1 ± 1.1	10.2 ± 1.7	10.0 ± 2.0
training time (h/month)	55.6 ± 3.3	51.1 ± 1.4	54.8 ± 2.8

K1M, kayak men; K1W, kayak women; C1M, canoe men; ± - mean ± standard deviation.

All athletes were members of the Slovak national team in junior or under 23 categories. The performance level of the athletes was set accordingly to the recommendation of McKay et al. (2022) The Ethical Committee of the Faculty of Physical Education and Sports, Comenius University in Bratislava, approved the study in accordance with the ethical standards of the Helsinki Declaration, and athletes were fully informed about the nature and possible risks of all procedures before providing written informed consent.

### Procedures

All the athletes took part in two flatwater testing sessions. Both test and retest sessions were conducted by one experienced examinator, on the artificial flatwater course without any streams, under a similar meteorological condition, to minimalized influence of these factors on the possible variations in the measurement. All athletes have previous practical experience with tests and were familiarized with testing procedure 1 week before first testing session. The instructions were identical in every test session. Each testing session was separated by a minimum of 3 days and a maximum of 5 days. To the minimize any influence of circadian variation all athletes performed their testing at the same time of the day. Athletes were asked to obtain enough sleep on the preceding night, not to participate in intense exercise in the 24 h before testing, and not to eat in the 2 h before the measurement session.

### Test protocols

#### Flatwater testing

Flatwater testing sessions were performed on an artificial canal with no water flow or underwater currents. During both test and retest testing sessions athletes performed 4 flatwater tests.

**FIGURE 1:**
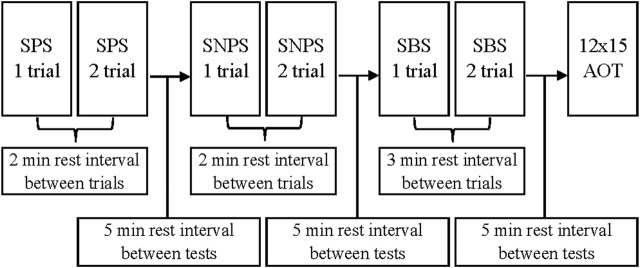
Testing procedure timeline by (Vajda and Piatrikova, 2022). Reproduced from Vajda and Piatrikova (2022), licensed under the International Journal of Sports Physiology and Performance © Human Kinetics.

• SPS—Sprints with a turn to the preferred side (2 × 15 m shuttle sprints)
• SNPS—Sprints with a turn to the non-preferred side (2 × 15 m shuttle sprints)
• SBS—Sprints with turns to both sides (2 × 15 m shuttle sprints, a figure of eight)
• 12 × 15 AOT—12 × 15 m all out shuttle test (6x a figure of eight). The timeline scheme of the testing procedure is described in Figure 1.

**FIGURE 2:**
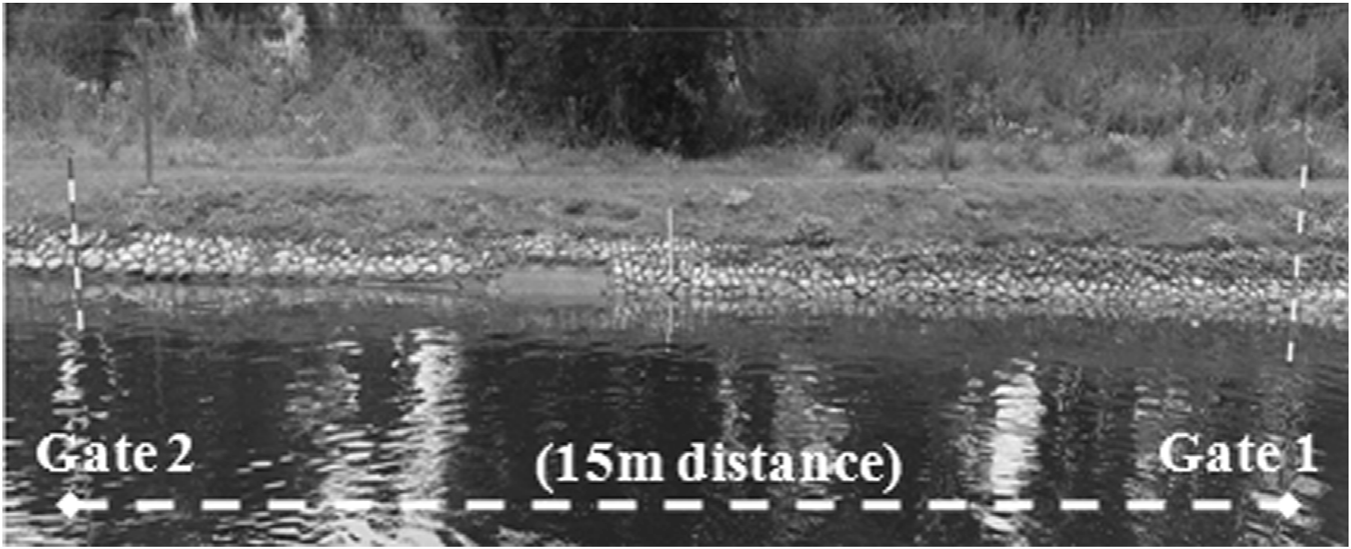
Representation of the course for all four flatwater tests by (Vajda and Piatrikova, 2022). Reproduced from Vajda and Piatrikova (2022), licensed under the International Journal of Sports Physiology and Performance © Human Kinetics.

The course consisted of 2 gates (gate 1 and gate 2) in the opposite position (see Figure 2). The distance between gates was 15 m.

All tests started from a stationary position with the top of the boat in line between poles (gate 1). Subjects were instructed to build up to their maximal velocity and maintain their highest paddling velocity throughout the entire test. Each athlete performed their own warm-up routine and test individually, so there was no racing/pacing with other individuals. These tests and testing sessions timeline were previously published by Vajda and Piatrikova (2022) and are described in Table 2.

**TABLE 2:** Detailed description of the flatwater tests by (Vajda and Piatrikova, 2022). Reproduced from Vajda and Piatrikova (2022), licensed under the International Journal of Sports Physiology and Performance © Human Kinetics.

**Tests**	**Test descriptions**	**Test visualization**	**Time measurement**
Sprints with a turn to the preferred side (SPS) (2 × 15 m shuttle sprints)	subjects sprinted from the starting gates to the opposite gate then turned to the preferred side and paddled back to the starting gate	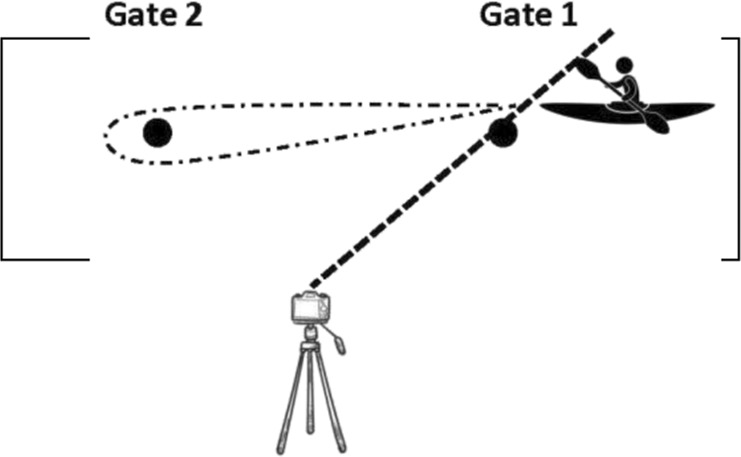	The time was started/stopped when the body of the subject crossed the pole line (gate 1)
Sprints with a turn to the non-preferred side (SNPS) (2 × 15 m shuttle sprints)	subjects sprinted from the starting gates to the opposite gate then turned to the non-preferred side and paddled back to the starting gate	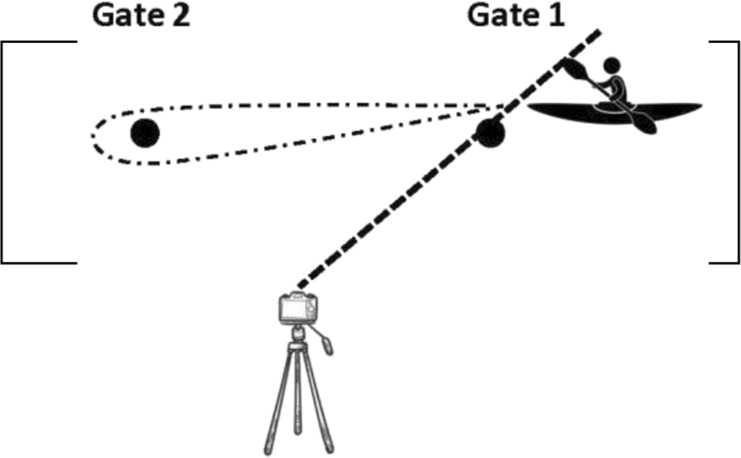	The time was started/stopped when the body of the subject crossed the pole line (gate 1)
Sprints with turns to both sides (SBS) (2 × 15 m shuttle sprints, a figure of eight)	subjects paddled from the starting gate to the opposite gate then turned to the left side, paddled back to the starting gate then turned to the right side and paddled to the opposite gate	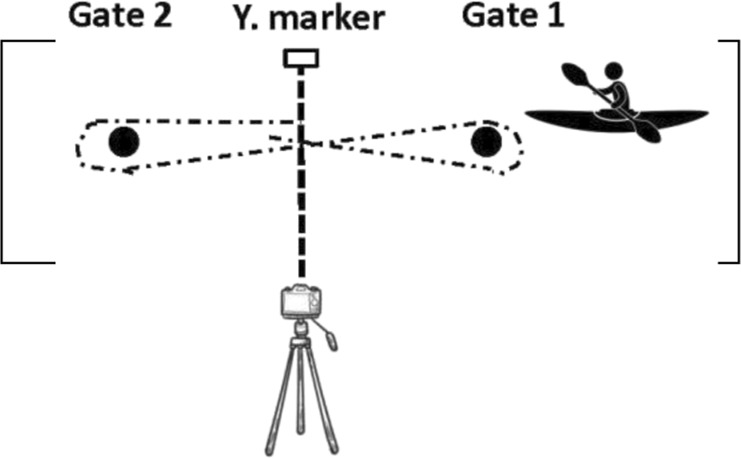	The time was started/stopped when the body of the subject crossed the yellow marker which was placed in the middle of the course (between gate 1 and gate 2)
12 × 15 m all out shuttle test (12 × 15 m AOT)	subjects paddled from the starting gate to the opposite gate then turned to the left side, paddled back to the starting gate then turned to the right side and paddled to the opposite gate and repeated this 6 times	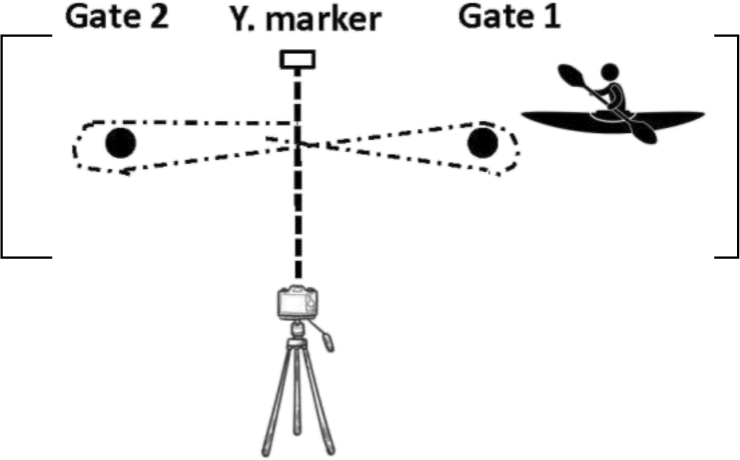	The time was started/stopped when the body of the subject crossed the yellow marker which was placed in the middle of the course (between gate 1 and gate 2)

Tests were recorded by a camera Panasonic HC-V800 with 60 fps (Panasonic EP-K, Osaka, Japan) placed on a tripod at a height of 125 cm and time was subsequently measured by video analyses software Dartfish Team Pro (Dartfish HQ, Fribourg, Switzerland). The attempt with the fastest time of each test was used for further analyses.

### Statistical analysis

All statistical procedures were performed using the SPSS 25 (IBM, New York, United States) and data are presented as mean ± standard deviations (SD) and 95% confidence interval (95%CI). All parameters were tested for normal distribution by the Shapiro-Wilks test for normality before further analysis. The paired samples *t*-test was used to calculate the differences between the values obtained in the two testing sessions. Statistical significance was accepted at *p* ≤ 0.05.

Test—retest relative reliability was assessed using the intraclass correlation coefficient (ICC) test and interpreted as follows: as poor (<0.5), moderate (0.5–0.75), good (0.76–0.9), and excellent (>0.9). Koo and Li (2016).

To assess absolute reliability, we calculated the standard error of measurement (SEM). The SEM represents the typical variability in an athlete’s time over multiple trials of this test. To obtain the SEM, the standard deviation of the difference scores between trial one and two was divided by the square root of two, for each test (Hopkins, 2000). A 95% confidence interval for the SEM was calculated using the chi-square distribution (Hopkins, 2000). The coefficient of variation (CV) was also calculated.

To more practically relevant results for coaches, we calculated the smallest worthwhile change (SWC). This is the change in time required between trials to be reasonably confident that a true change has occurred for the athlete, rather than a change due to random error. To obtain the SWC, we used a simple metric, the SEM multiplied by a factor of 1.5 (Hopkins, 2000). The SEM and SWC analyses were completed in Excel (Microsoft, Redmond, USA). Pearson correlation analysis was used to assess the linear correlation of the data from the two tests. The relationship was assessed as: small (*r* = 0.1–0.3), moderate (*r* = 0.3–0.5), large (*r* = 0.5–0.7), very large (*r* = 0.7–0.9), nearly perfect (*r* > 0.9) (Hopkins et al., 2009).

## Results

All 22 athletes completed the four tests on each of the two test days and no data were excluded from the analyses. Completion times for the four flatwater tests are in Table 3 for each category. The fastest times were achieved by the K1M, followed by the K1W, and lastly the C1M. 95% of athletes within their respective categories were within one second of each other’s times for the SPS and SNPS tests, with the K1M exhibiting the lowest variability.

**TABLE 3:** Descriptive parameters derived from flatwater tests and sessions.

Flat-water tests		K1M	K1W	C1M	Pooled
		(n = 8)	(n = 7)	(n = 7)	(n = 22)
**SPS test (s)**	X±SD	11.39 ± 0.14	13.08 ± 0.42	13.67 ± 0.52	12.65 ± 1.07
	CI95%	11.26–11.51	12.69–13.47	13.19–14.16	12.17–13.13
**SPS retest (s)**	X±SD	11.36 ± 0.20	13.00 ± 0.38	13.59 ± 0.57	12.59 ± 1.05
	CI95%	11.18–11.53	12.65–13.35	13.06–14.13	12.12–13.06
**SNPS test (s)**	X±SD	11.58 ± 0.14	13.24 ± 0.47	14.08 ± 0.62	12.90 ± 1.15
	CI95%	11.46–11.70	12.80–13.68	13.50–14.66	12.39–13.31
**SNPS retest (s)**	X±SD	11.60 ± 0.21	13.31 ± 0.48	14.05 ± 0.53	12.92 ± 1.14
	CI95%	11.42–11.78	12.86–13.75	13.55–14.55	12.42–13.43
**SBS test (s)**	X±SD	13.53 ± 0.25	15.31 ± 0.75	15.86 ± 0.74	14.84 ± 1.18
	CI95%	13.32–13.50	14.61–16.00	15.17–16.55	14.31–15.37
**SBS retest (s)**	X±SD	13.59 ± 0.23	15.38 ± 0.80	15.90 ± 0.61	14.90 ± 1.17
	CI95%	13.40–13.78	14.64–16.13	15.34–16.47	14.38–15.41
**12 × 15m AOT test (s)**	X±SD	93.89 ± 2.20	103.16 ± 2.94	104.36 ± 3.67	100.11 ± 5.70
	CI95%	91.89–95.57	100.43–105.89	100.96–107.75	97.58–102.64
**12 × 15m AOT retest (s)**	X±SD	93.46 ± 1.56	103.31 ± 2.81	104.13 ± 3.78	99.99 ± 5.72
	CI95%	92.15–94.76	100.71–105.91	100.63–107.63	97.45–102.53

K1M, kayak men; K1W, kayak women; C1M, canoe men; SPS, sprints with a turn to the preferred side; SNPS, sprints with a turn to the non-preferred side; SBS, sprints with turns to both sides; 12 × 15 m AOT, all-out shuttle test, X±SD, mean±standard deviation; CI95%–95% of confidence interval.

Relative reliability is in Table 4. All four flatwater tests had excellent relative reliability demonstrated by ICC values 0.96 or greater. Thus, athlete rankings were well maintained between the two trials.

**TABLE 4:** Relative reliability of flatwater tests.

Flat-water tests	Test session	Retest session	ICC (relative reliability)	CI (95%) of ICC	p
**SPS (s)**	12.65 ± 1.07	12.59 ± 1.05	0.98	0.95–0.99	0.174
**SNPS (s)**	12.90 ± 1.15	12.92 ± 1.14	0.97	0.94–0.99	0.717
**SBS (s)**	14.84 ± 1.18	14.90 ± 1.17	0.98	0.97–0.99	0.164
**12 × 15m AOT (s)**	100.11 ± 5.70	99.99 ± 5.72	0.96	0.92–0.98	0.689

SPS, sprints with a turn to the preferred side; SNPS, sprints with a turn to the non-preferred side; SBS, sprints with turns to both sides; 12 × 15 m AOT, all-out shuttle test, mean ± standard deviation; s = seconds, ICC, intra-class coefficient, CI95% = 95% of confidence interval of ICC, p = paired sample test significance.

Absolute reliability is in Table 5. The test with the lowest SEM was SBS, where athletes tended to vary by 0.13 s between trials. Even though the SBS test was longer than either of the tests with only one turn, the absolute reliability was higher. This is evident by the lowest CV for the SBS test. This also meant that the SBS test required the smallest change in time to be considered a real improvement, with a SWC of 0.19 s. Naturally, as the 12 × 15 m AOT was the longest test, the SEM was highest for the 12 × 15 m AOT, at 1.05 s. However, the CV was comparable to the other tests. The SWC for this test was 1.58 s, meaning that athletes who complete two trials of the 12 × 15 m AOT need to improve by at least 1.58 s to be reasonably confident that a real improvement occurred.

**TABLE 5:** Absolute reliability of flatwater tests.

**Flat-water tests**	**SEM (s)**	**CI (95%) (s)**	**SWC (s)**	**CV (%)**
**SPS (s)**	0.14	0.11–0.20	0.21	1.1
**SNPS (s)**	0.18	0.14–0.25	0.26	1.4
**SBS (s)**	0.13	0.10–0.19	0.19	0.9
**12 × 15m AOT (s)**	1.05	0.81–1.51	1.58	1.1

SPS, sprints with a turn to the preferred side; SNPS, sprints with a turn to the non-preferred side; SBS, sprints with turns to both sides; 12 × 15 m AOT, all-out shuttle test; s = seconds, SEM, standard error of measurement; SWC, smallest worthwhile change.

Correlations between the four tests are in Table 6. All correlations were nearly perfect. The highest correlations were between the SPS and SNPS tests.

**TABLE 6:** Pearson correlations between tests and sessions.

Flat-water tests	SPS retest (s)	SNPS test (s)	SNPS retest (s)	SBS test (s)	SBS retest (s)	12 × 15 m AOT test (s)	12 × 15 m AOT retest (s)
**SPS test (s)**	–	0.991**	0.975**	0.960**	0.953**	0.922**	0.949**
**SPS retest (s)**		0.979**	0.976**	0.954**	0.957**	0.933**	0.951**
**SNPS test (s)**			–	0.953**	0.947**	0.917**	0.930**
**SNPS retest (s)**				0.937**	0.947**	0.924**	0.938**
**SBS test (s)**					–	0.940**	0.960**
**SBS retest (s)**						0.959**	0.964**
**12 × 15m AOT test (s)**							–

SPS, sprints with a turn to the preferred side; SNPS, sprints with a turn to the non-preferred side; SBS, sprints with a turns to both sides; 12 × 15 m AOT, all-out shuttle test, s = session, **p < 0.001. Correlations were calculated using ICC, instead of Pearson correlation for each test compared to the same test retest.

## Discussion

To the best of our knowledge, this is the second study realized test-retest analyses of performance-related tests on flatwater tests in canoe slalom. The first one was published by Baláš et al. (2020) who realized test-retest measurement of three flatwater tests of different distances (40, 80, and 200 m) with turning maneuvers. The authors found that these tests provide moderate to excellent reliability (ICC = 0.68–0.92). In the present study, among international-level canoe slalom athletes, rankings between test and retest were well preserved for all four tests, demonstrating excellent relative reliability (ICC = 0.96–0.98) (see Table 4.). The typical variation in test times did not exceed 1.4% across the four tests, demonstrating excellent absolute reliability (see Table 5.).

Accordingly, to study of Baláš et al. (2020) it should be noted that the authors did not use regular turning maneuvers around the poles but used pivoting which is a common maneuver of a canoe slalom training routine but not regularly performed in competition runs. Also, authors did not use turning around the gates, but athletes performed turns on marked points. Accordingly, to this information and our results, we propose our tests to be more appropriate to analyze performance-related physical fitness in canoe slalom.

Coaches should take particular note of the values for the SEM and SWC of these four tests. When flatwater tests are used to assess changes in performance-related physical fitness, it is imperative that variability in the test is not interpreted as a real improvement or deterioration by the athlete. Athletes in the SPS, SNPS, and SBS typically varied by 0.13–0.18 s between attempts (see Table 5.), and this variability is probably not strongly affected by systematic change in training status, fatigue, or nutrition. Coaches can rely on the SWC values to assess whether a real improvement or deterioration has occurred for their athletes over the course of training. For example, in the SPS, SNPS, and SBS, athletes must improve their times by at least 0.19–0.26 s in order to attribute this change to an improvement due to training (or the opposite for a deterioration). Because the 12 × 15 m AOT is a longer test, this SWC is larger. Athletes must improve by at least 1.58 s to conclude that their improvement is a real effect due to training. Based on the unpublish data of authors (harvested over 2019-2022) changes of performance in 12 × 15 m AOT may vary 1.9–5.2 s across the training season, making this SWC quite good.

The four flatwater tests here provide an opportunity to assess different aspects of canoe slalom performance. The SBS test demonstrated the highest relative and absolute reliability. Previously, we also found the SBS had greater validity than the SPS and SNPS tests when comparing rankings between these flatwater tests and international-level competition venues. Vajda and Piatrikova (2022) Thus, we highly recommended assessing changes in performance-related physical fitness of athletes using the SBS test. It is also useful to conduct the SPS and SNPS tests, as it is through these tests that any asymmetry between preferred and non-preferred turning sides can be assessed and possibly targeted for improvement. For example, coaches may compare their athlete’s asymmetry to that exhibited by the international-level athletes in this study—if their athlete demonstrates greater asymmetry than the majority of athletes in this study, it is an area they can target in training for improvement. The 12 × 15 m AOT completes the testing battery by assessing athlete fatigue over a time similar to that of competition. We have shown in this study the 12 × 15 m AOT is a reliable test, and our previous work, showed the 12 × 15 m AOT had the highest validity when comparing 12 × 15 m AOT rankings to rankings on harder whitewater courses of the same difficulty as international competitions. Vajda and Piatrikova (2022).

This study has couple of limitations. We only recruited the athletes from one of K1M, K1W, and athletes from the C1W category were not included in the study our findings cannot be generalized to the C1W category. Also, the performance level of recruited athletes varies from international level (most of the athletes) to world-class athletes (few athletes). For future research it would be appropriate to harvest additional data during these tests using accelerometers (to measure velocities, acceleration, and deceleration), mobile spiro ergometer device (to collect cardiorespiratory parameters) as well as an instrumented paddle with tensometers (to measure force production during the propulsive phase of the strokes and side asymmetries).

## Conclusion

The results obtained from this study have shown the excellent test-retest reliability of all four flatwater tests (ICC = 0.96–0.98). Additionally, results have shown SEM (SPS: 0.14; SPNPS 0.18; SBS: 0.13 and 12 × 15 m AOT: 1.05) and SWC (SPS: 0.21; SPNPS 0.26; SBS: 0.19 and 12 × 15 m AOT: 1.58) which are important information for coaches and other researchers who will use these tests in the future. Based on our results we suggest to coaches use these valid and reliable flatwater tests to assess changes in performance-related physical fitness of their athletes over time, to verify the effectiveness of training programs focused on improvement in specific physical fitness of athletes as well as to identify asymmetries between the preferred and non-preferred side in canoe slalom athletes.

## Data Availability

The original contributions presented in the study are included in the article/Supplementary material, further inquiries can be directed to the corresponding author.
